# Epithelial Dual Oxidase 2 Shapes the Mucosal Microbiome and Contributes to Inflammatory Susceptibility

**DOI:** 10.3390/antiox12101889

**Published:** 2023-10-21

**Authors:** Juan Camilo Castrillón-Betancur, Víctor Alonso López-Agudelo, Nina Sommer, Sven Cleeves, Joana Pimenta Bernardes, Saskia Weber-Stiehl, Philip Rosenstiel, Felix Sommer

**Affiliations:** 1 Institute of Clinical Molecular Biology, University of Kiel, Rosalind-Franklin-Straße 12, 24105 Kiel, Germany; 2 Fraunhofer Institute for Toxicology and Experimental Medicine ITEM, Nikolai-Fuchs-Straße 1, 30625 Hannover, Germany

**Keywords:** inflammation, DUOX2, ROS, microbiome

## Abstract

Reactive oxygen species (ROS) are highly reactive molecules formed from diatomic oxygen. They act as cellular signals, exert antibiotic activity towards invading microorganisms, but can also damage host cells. Dual oxidase 2 (DUOX2) is the main ROS-producing enzyme in the intestine, regulated by cues of the commensal microbiota and functions in pathogen defense. DUOX2 plays multiple roles in different organs and cell types, complicating the functional analysis using systemic deletion models. Here, we interrogate the precise role of epithelial DUOX2 for intestinal homeostasis and host-microbiome interactions. Conditional *Duox2*^∆IEC^ mice lacking DUOX2, specifically in intestinal epithelial cells, were generated, and their intestinal mucosal immune phenotype and microbiome were analyzed. Inflammatory susceptibility was evaluated by challenging *Duox2*^∆IEC^ mice in the dextran sodium sulfate (DSS) colitis model. DUOX2-microbiome interactions in humans were investigated by paired analyses of mucosal DUOX2 expression and fecal microbiome data in patients with intestinal inflammation. Under unchallenged conditions, we did not observe any obvious phenotype of *Duox2*^∆IEC^ mice, although intestinal epithelial ROS production was drastically decreased, and the mucosal microbiome composition was altered. When challenged with DSS, *Duox2*^∆IEC^ mice were protected from colitis, possibly by inhibiting ROS-mediated damage and fostering epithelial regenerative responses. Finally, in patients with intestinal inflammation, DUOX2 expression was increased in inflamed tissue, and high DUOX2 levels were linked to a dysbiotic microbiome. Our findings demonstrate that bidirectional DUOX2-microbiome interactions contribute to mucosal homeostasis, and their dysregulation may drive disease development, thus highlighting this axis as a therapeutic target to treat intestinal inflammation.

## 1. Introduction

Inflammatory bowel disease (IBD) is a human disorder characterized by chronic inflammation of the digestive tract, including Crohn’s disease (CD) and ulcerative colitis (UC). The gut microbiome, the community of microorganisms, plays a significant role in the development and progression of IBD. A dysfunctional microbiome may trigger an abnormal immune response and is thought to contribute to chronic inflammation and damage to the intestinal lining [1]. The intestinal epithelium serves as a barrier, keeping commensal and pathogenic microorganisms in check. Several mucosal defense mechanisms are in place, including the release of mucus, antimicrobial peptides, secretory IgA, or the production of reactive oxygen species (ROS). ROS are highly reactive molecules with antibiotic properties, and therefore, eukaryotes employ ROS as protective components of innate immunity [2,3]. However, excessive ROS production can also cause DNA damage, oxidative stress, or cell death. Therefore, ROS production needs to be tightly regulated. Excessive ROS production has been linked to various diseases, including IBD [4]. Significant levels of ROS can be produced by intestinal epithelial cells (IECs) in response to bacterial infection in the intestinal tract. ROS are either formed as byproducts of cellular energy metabolism but also through the action of dedicated ROS-producing enzymes of the NADPH oxidase (NOX) family [5,6], which can be subdivided into two classes: NOX and DUOX (dual oxidase) proteins. DUOX2 is the most highly expressed member of the NOX family within the intestinal epithelium [7,8,9,10] (Figure S1). DUOX2 is a transmembrane protein producing H_2_O_2_ by the transfer of two electrons from NADPH to O_2_, which is dependent on Ca^2+^ availability and an association with its maturation factor, DUOXA2 [11]. Several lines of evidence suggest an important function of DUOX2 in epithelial innate immunity, control of the microbiome, and regenerative responses of the epithelium. DUOX2-generated H_2_O_2_ limits *Helicobacter* infection [12], and the deletion of DUOX2 activity leads to increased uptake of bacterial material, resulting in a pro-inflammatory milieu within the intestinal mucosa [9]. ROS production by DUOX2 is induced by the intracellular innate immune receptor NOD2 [13]. DUOX2 is required for *Citrobacter rodentium*-dependent induction of pro-inflammatory Th17 cell differentiation [14]. Importantly, deleterious DUOX2 variants are associated with dysregulated microbiome-immune interactions and an increased risk of developing IBD [15,16]. However, DUOX2 is not yet reported as a risk factor for IBD in adult patients, for example, by genome-wide association studies (GWAS) [15,17], and mice with non-functional DUOX1 and DUOX2 by deletion of their maturation factors DUOXA1 and DUOXA2 in the entire body did not display an altered colitis susceptibility [9]. Due to these potentially conflicting data, the precise role of DUOX2 for immune homeostasis and host-microbiome interactions during health and disease remained unclear. Therefore, we aimed to elucidate the function of epithelial DUOX2 for intestinal homeostasis. To that end, we generated mice lacking DUOX2 specifically in IECs using the CRE-lox system in combination with the IEC-specific *Villin* promoter-termed *Duox2*^fl/fl^-Villin::*Cre*^+^ or *Duox2*^∆IEC^ or “DX2” mice. Here we show that (1) unchallenged *Duox2*^∆IEC^ mice did not display any obvious intestinal phenotype but harbored an altered mucosal microbiome, that (2) loss of epithelial DUOX2 protected from dextran sodium sulfate (DSS)-induced colitis associated with enhanced epithelial regenerative responses and fecal microbiome changes, and that (3) dysregulated DUOX2 expression in the mucosa of IBD patients coincided with an altered microbiome.

## 2. Materials and Methods

### 2.1. Animals

*Duox2*^fl/fl^ mice were generated from C57/BL6N embryonic stem cells of the KOMP repository (*Duox2*^tm1a(KOMP)Wtsi^) using a conditional-ready exon trapping strategy. In brief, the neomycin selection cassette was inserted at position 122121456 of Chromosome 2 (mm37). The cassette contained an FRT site followed by a lacZ sequence a loxP-flanked neomycin resistance gene with an internal FRT site. A third loxP site was inserted downstream of the targeted exons at position 122119441 (Figure S2A). Mice carrying the allele were obtained by standard procedures using the same genetic background at Taconic (Germantown, NY, USA). These *tm1a* offspring were bred with an flp deleter strain to remove the neomycin selection cassette and generate the final conditional allele *tm1c* (Figure S2A). To create the *Duox2*^∆IEC^ mouse line, we crossed the conditional *tm1c* carrying floxed *Duox2* alleles with mice expressing the CRE recombinase under the control of the IEC-specific *Villin* promoter. As controls, we used littermate *Duox2*^fl/fl^ mice not carrying any CRE recombinase, referred to as WT mice.

All animal experiments were approved by the local animal safety review board of the federal ministry of Schleswig Holstein and conducted according to national and international laws and policies (V 312-72241.121-33 (95-8/11) and V242-62324/2016 (97-8/16)). Specific-pathogen-free (SPF) animals were housed in the Central Animal Facility (ZTH) of the University Hospital Schleswig Holstein (UKSH, Kiel, Germany).

All mice were kept under a 12 h light cycle and fed a gamma-irradiated diet ad libitum. Mice were killed by cervical dislocation prior to removing tissues for histological and molecular analyses. For basal phenotyping, we used 9 to 12-week-old male and female mice. For DSS-induced colitis, we used 10 to 14-week-old male mice. Both genotypes were co-housed throughout the experiments. Mice received 2% (*w*/*v*) DSS in autoclaved tap water to induce colitis. Disease activity was determined by a combination of weight loss (0 = 0–5%, 1 = 5–10%, 2 = 10–15%, 3 = 15–20% and 4 = >20%), stool consistency (0 = formed, 1 = formed but soft, 2 = unformed, 4 = diarrhea) and rectal bleeding (0 = negative Haemoccult (Beckman Coulter), 1 = slightly positive Haemoccult, 2 = strongly positive Haemoccult, 3 = visible blood on stool, 4 = rectal bleeding) as previously described [18].

### 2.2. Isolation of Primary Cells

IECs were isolated from intestinal tissue using the Lamina Propria Dissociation Kit (Miltenyi BioTech, Bergisch Gladbach, Germany) according to the manufacturer’s protocol with minor deviations as described before [19]. In brief, intestinal epithelial cells were isolated by disruption of the structural integrity of the epithelium using ethylenediaminetetraacetic acid (EDTA) and dithiothreitol (DTT). The purity of individual IEC fractions was analyzed by flow cytometry on a FACS Calibur flow cytometer (B&D, Heidelberg, Germany) with Cellquest analysis software version 5.1 (B&D, Heidelberg, Germany). We used the Anti-EpCam-PE (Clone: G8.8, Biolegend, San Diego, CA, USA) antibody for analysis of IEC purity. FACS data was analyzed using Flowing Software version 2.5.1 (Perttu Terho, Turku Centre for Biotechnology, Finland).

### 2.3. Isolation and Culture of Intestinal Organoids

Intestinal organoids were generated following procedures described earlier by Sato et al., 2009 [20]. Organoids were cultivated in 24 well plates in Matrigel (BD) with ENR-conditioned medium supplemented with 0.1% human recombinant EGF (50 µg/mL) as described by Sato et al., 2011 [21]. ENR-conditioned medium consisted of 70% (*v*/*v*) 2 × basal medium (Advanced DMEM/F12 supplemented with HEPES [1M], Glutamax [100×], Penicillin/streptomycin 10,000 U/mL [1:50] and N-Acetylcysteine [500 mM]), 10% (*v*/*v*) Noggin-conditioned medium and 20% R-Spondin-conditioned medium. To generate a 2D organoid culture, intestinal organoids (3D) were pelleted and resuspended with TrypLE Express (Invitrogen, Carlsbad, CA, USA) and incubated for 6 min at 37 °C. In this condition, organoids were completely dissociated into single cells. Cells were counted and seeded at a concentration between 50 and 100 × 10^3^ cells/well in a 96-well plate precoated with Matrigel in 100 µL/well medium containing Y-27632 (10 μmol/L; Sigma-Aldrich, St. Louis, MO, USA).

### 2.4. ROS Assay

H_2_O_2_ released from organoids monolayer to the medium was measured with ROS-Glo™ H_2_O_2_ Assay (Promega, Madison, WI, USA) following the manufacturer’s protocol. All measurements were corrected for autofluorescence of the medium. To account for differences in the cell numbers, the H_2_O_2_ production was normalized to the total DNA content of the incubated organoid monolayer and expressed in luminescence per ng of DNA.

### 2.5. Organoid Proliferation Assay

To quantify the growth of organoids in relation to DUOX2 status, 1 × 10^4^ organoid cells/well were seeded in a 96-well plate as described before and incubated at 37 °C and 5% CO_2_. After 4 days, the area of each 2D organoid colony was visualized using a Zeiss Axiovert Observer A1 inverted microscope (Zeiss, Jena, Germany) and quantified using ZEN pro version 3.4 (Zeiss, Jena, Germany) software.

### 2.6. RNA Isolation and qPCR

Total RNA was extracted using the RNeasy Mini Kit (Qiagen, Hilden, Germany) according to the manufacturer’s protocol. RNA concentration was measured using a NanoDrop ND-1000 spectrophotometer (PeqLab Biotechnologie, Erlangen, Germany). A total of 1 µg of total RNA was reverse transcribed to cDNA using the Maxima H Minus First Strand cDNA Synthesis Kit (ThermoFisher Scientific, Waltham, MA, USA). qPCR was carried out using SYBR Select Master Mix (Applied Biosystems, Waltham, MA, USA) according to the manufacturer’s instructions on a Viia 7 Real-Time PCR System (ThermoFisher Scientific). Expression levels were normalized to *Actb* (β-actin). Primer sequences for qPCR are listed in Table S1.

### 2.7. Microbiome Analysis Using 16S Amplicon Sequencing

Intestinal “mucosa” and “lumen” samples were prepared by excision of a corresponding tissue segment followed by gently pressing out the content (“lumen” fraction) and washing of the remaining tissue specimen (“mucosa”) by thorough flushing with sterile PBS. All samples were immediately frozen in liquid nitrogen and stored at −80 °C until further analyses. DNA was isolated from intestinal material using the DNeasy PowerSoil Kit (Qiagen) following the manufacturer’s protocol. Extracted DNA was eluted from the spin filter silica membrane with 100 µL of elution buffer and stored at −80 °C. MiSeq sequencing and 16S profiling was performed as described earlier [22,23], with the following modifications: the V3-V4 region of the 16S gene was amplified using the dual barcoded primers 319F (ACTCCTACGGGAGGCAGCAG) and 806R (GGACTACHVGGGTWTCTAAT) [24]. Each primer contained additional sequences for a 12-base Golay barcode, an Illumina adaptor, and a linker sequence [25]. PCR was performed using the Phusion Hot Start Flex 2× Master Mix (NEB) in a GeneAmp PCR system 9700 (Applied Biosystems) and the following program (98 °C for 3 min, 25–30× (98 °C for 20 s, 55 °C for 30 s, 72 °C for 45 s), 72 °C for 10 min, hold at 4 °C). Performance of the PCR reactions was checked using agarose gel electrophoresis. Normalization was performed using the SequalPrep Normalization Plate Kit (Thermo Fisher Scientific, Darmstadt, Germany) following the manufacturer’s instructions. Equal volumes of SequalPrep-normalized amplicons were pooled and sequenced on an Illumina MiSeq (2 × 300 nt).

MiSeq 16S amplicon sequence data were processed using DADA2 [26] workflow (https://benjjneb.github.io/dada2/bigdata.html accessed on 25 October 2022) with default parameters resulting in abundance tables of amplicon sequence variants (ASVs). Taxonomy was assigned using the Bayesian classifier provided in DADA2 and using the Silva rRNA database v.138 [27]. Samples with >5000 reads were retained for analyses. Uni- and multivariate analyses of the 16S data were conducted in the R statistical software (v.4.2.1) under phyloseq [28] (v.1.40.0), vegan [29] (v.2.6-2) and MAasLin2 [30] (v.1.10.0). All samples for diversity analyses were normalized by rarefaction to the minimum shared read count to account for differential sequencing depth among samples. Relative abundance was calculated by dividing the number of reads for an ASV by the total number of sequences in the sample. Alpha diversity measures and beta diversity were computed using Bray–Curtis, and differences were visualized in a principal coordinate analysis plot. Constraint analysis of principal coordinates was computed on the DSS experiment data based on Aitchison distances. Associations of microbiome composition to specified covariates were tested with the implementation of PERMANOVA models (using the adonis2 function from the vegan package). The P and R^2^ values were determined by 10,000 permutations. Linear discriminant analysis (LDA) effect size (LEfSe) [28] was performed using the online tool available at http://huttenhower.sph.harvard.edu/galaxy accessed between 1 October 2022 and 1 December 2022. LDA denotes taxa based on their contribution to the overall observed differences between groups, i.e., taxa being significantly increased in abundance. To detect differences in changes in microbial features between WT and DX2 over time or among mucosal or luminal small intestine or colon tissue, we built linear mixed models using the MaAslin2 package [30] in previously wrench normalized abundances [31]. The model included time and/or genotype group and individual mice as a random variable. *p* values were corrected for multiple hypothesis testing using the Benjamin-Hochberg procedure, and a false discovery rate < 0.05 was defined as the significant threshold. Only features appearing in at least 20% of the samples were included in the analysis.

To explore the association between intestinal mucosal *DUOX2* expression and the abundance of microbial taxa in the fecal microbiome of IBD patients, we retrieved publicly available biopsy transcriptomics and stool metagenomics data of the Human Microbiome Project Phase 2 (HMP2) [32] from ibdmdb.org. Transcriptomics data from intestinal biopsies were normalized by TPM and log2 transformed. Patients were classified into high/low *DUOX2* expression groups according to the median *DUOX2* expression of all IBD patients. To then detect associations in changes of microbial features with levels of *DUOX2* expression, we built a linear mixed model on species and genus relative abundances of patients with matched transcriptomics and stool metagenomics (*n* = 50). The model included DUOX2 expression (high or low) as a fixed effect variable.

### 2.8. Western Blot Analyses

Intestinal organoids derived from *Duox2*^∆IEC^ and littermate control mice were lysed using RIPA buffer + 1% Halt Protease inhibitor cocktail (Thermo Fisher Scientific). Lysates were heated to 95 °C for 5 min and centrifuged at 16,000× *g* for 15 min at 4 °C to remove cell remnants. Protein concentrations were measured by DC Protein Assay (BioRad, Hercules, CA, USA) according to the manufacturer’s protocol. Equal amounts of lysates containing Laemmli buffer (250 mM TRIS, 10% (*v*/*v*) SDS, 50% (*v*/*v*) Glycerol, 500 mM DTT and Bromophenol blue) were heated at 95 °C and electrophoresed on 10% or 15% polyacrylamide gels under standard SDS-PAGE conditions before being transferred onto an immuno-Blot PVDF Membrane (BioRad). Protein-loaded membranes were blocked with 5% (*w*/*v*) non-fat dry milk or bovine serum albumin (BSA) in Tris-buffered saline (TBS) (200 mM TRIS, 1.37 M NaCl) supplemented with 0,1% (*v*/*v*) Tween 20 for 1 h, incubated with primary antibody (mouse anti-DUOX2, Millipore #MABN787, mouse anti-Vinculin, Abcam #ab18058) overnight, washed three times with TBS-Tween-20 and then incubated with the secondary horseradish peroxidase (HRP)-conjugated antibody for 1 h at room temperature. Proteins were detected using the Pierce ECL Plus Substrate Kit (ThermoFisher).

### 2.9. Histology and Immunostaining

Tissue specimens were fixed in 10% formalin solution overnight at 4 °C and then embedded in paraffin. A total of 5 µm thick sections were cut and stained with hematoxylin and eosin (H&E) or subjected to immunostaining using the Vectastain ABC kit (Vector Laboratories, Newark, CA, USA), including antigen retrieval in boiling citrate buffer. Primary antibodies were incubated overnight. For immunostaining of DUOX2, we used a 1:250 diluted antibody (Millipore #MABN787). For immunostaining of Ki67, we used a 1:500 diluted mouse anti-Ki67 antibody (B&D, Heidelberg, Germany, cat. no. 556003). Slides were visualized using a Zeiss Imager Z1 microscope (Zeiss), and pictures were taken using ZEN pro (Zeiss) software. Histological disease activity was assessed as previously described [33].

### 2.10. ELISA

An enzyme-linked immunosorbent assay (ELISA) was used to detect cytokine levels in the serum of mice. For the measurement of murine CXCL1/KC, we used the Murine CXCL1/KC Quantikine (R&D Systems, Minneapolis, MO, USA), following the manufacturer’s protocol. The plates were coated overnight with an antigen-specific capture antibody at room temperature. Afterward, the plates were washed with 0.05% Tween 20 in DBPS and blocked using 1% BSA in DBPS. A streptavidin-bound horse radish peroxidase antibody was used as a secondary antibody following the manufacturer’s protocol. The absorbance was measured with an Infinite M200 Pro microplate reader (Tecan, Männedorf, Switzerland) using the Tecan-i.control software 1.9 (Tecan, Männedorf, Switzerland).

### 2.11. Data and Code Availability

The 16S amplicon sequencing data are accessible through the European Nucleotide Archive (ENA, https://www.ebi.ac.uk/ena accessed on 22 June 2023) under the accession number PRJEB63207. Additional data supporting the findings of this study, as well as all codes used to generate the bioinformatic analyses, are available from the corresponding author upon reasonable request.

### 2.12. Statistics Analysis

Biostatistical analyses were performed using GraphPad Prism (version 9) software (GraphPad, Inc., La Jolla, CA, USA) and R (v 3.2.5). Data from two groups were analyzed using an unpaired Student’s *t*-test, and comparisons between more than two groups were performed using one-way and two-way analysis of variance (ANOVA). * *p* < 0.05, ** *p* < 0.01, *** *p* < 0.001 and **** *p* < 0.0001. Data are presented as mean ± SEM.

## 3. Results

### 3.1. Epithelial DUOX2 Functions in Intestinal Homeostasis

**Figure 1:**
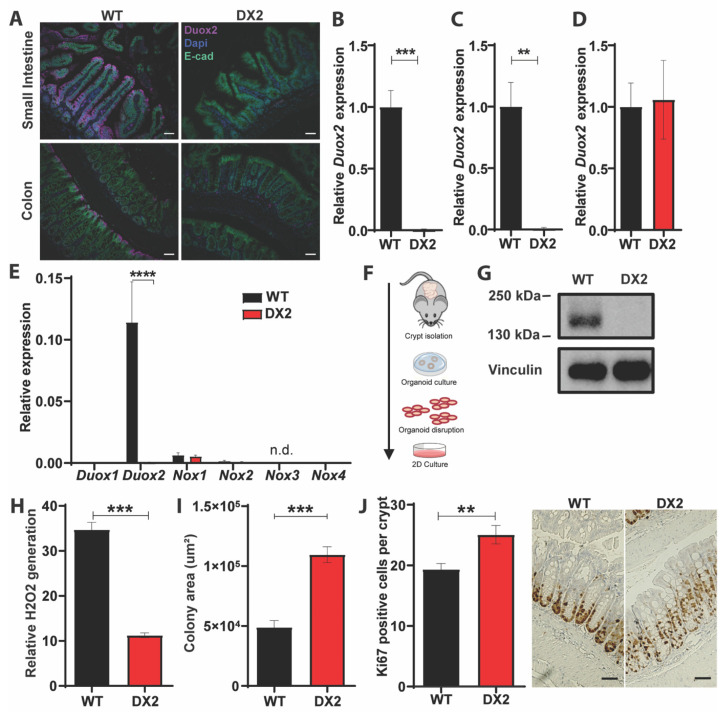
**DUOX2 controls epithelial proliferation and ROS production**. (**A**) The DUOX2 protein is located in the apical epithelium in the small and large intestines and the upper crypt in the small intestine. DUOX2 was completely absent in epithelial cells of *Duox2*^∆IEC^ mice. (**B**–**D**) Relative *Duox2* expression in small intestine (*n* = 7 per group) (**B**), colon (*n* = 9 for WT and *n* = 6 for DX2) (**C**) and liver (*n* = *n* = 5 for WT and *n* = 4 for DX2) (**D**) tissue of WT and *Duox2*^∆IEC^ mice. *Duox2* expression was abolished, specifically in intestinal tissues. (**E**) Relative expression of NOX family members in small intestinal IECs isolated from WT and *Duox2*^∆IEC^ mice (*n* = 8 per group). n.d. = not detected. (**F**) Schematic depiction of the procedure of intestinal organoid isolation and monolayer growth. (**G**) DUOX2 protein is not detectable in lysates of intestinal organoids derived from *Duox2*^∆IEC^ mice by Western blot. (**H**) Reduced H_2_O_2_ production in intestinal organoids derived from *Duox2*^∆IEC^ mice. H_2_O_2_ concentrations were normalized to DNA content to account for variation in cell density (*n* = 12 per group). (**I**) Accelerated growth of DUOX2-deficient intestinal organoids as measured by monolayer size (*n* = 5 per group). (**J**) Increased epithelial proliferation in *Duox2*^∆IEC^ mice as measured by Ki-67 positive cells per colon crypt (*n* = 8 per group). Scale bars correspond to 50 µm. ** *p* < 0.01, *** *p* < 0.001 and **** *p* < 0.0001.

To determine the role of epithelial DUOX2 for intestinal homeostasis, host-microbiome interactions, and inflammatory responses, we generated *Duox2*^fl/fl^-Villin::*Cre*^+^ mice lacking DUOX2 specifically in IEC, hereinafter referred to as *Duox2*^∆IEC^ or “DX2” mice. The *Duox2* gene was targeted by flanking exons 7–10 with loxP sites (Figure S2A), which are deleted by CRE recombinase yielding transcript encoding a non-functional DUOX2 protein. We used littermate *Duox2*^fl/fl^ mice, hereinafter referred to as WT mice, as controls. Successful conditional deletion of DUOX2 only in IECs was confirmed using three approaches. (i) Using immunofluorescence microscopy on intestinal sections, we found that in WT mice, the DUOX2 protein is predominantly present in differentiated enterocytes of the apical epithelium and to a lesser extent in epithelial cells of the crypt and lower villus (Figure 1A). In *Duox2*^∆IEC^ mice, no DUOX2 protein could be detected in IECs, but only very low levels were detectable in non-epithelial cells of the interstitium or submucosa. (ii) Using qPCR, we demonstrated that *Duox2* expression was robustly reduced in the small intestine and colon of *Duox2*^∆IEC^ mice compared to WT littermate controls, whereas *Duox2* expression was unaltered in the liver due to tissue specificity of the Villin::*Cre* system (Figure 1B–D). We also isolated small intestinal IECs and surveyed expressions of all NOX family members to check for the Duox2 deletion and test for compensatory effects on other ROS-producing NOX enzymes. In isolated IECs, we could not detect any *Duox2* expression, and no other NOX gene displayed any upregulation (Figure 1E). We could only detect low expression levels of *Nox1* and *Nox2* (18× and 72× lower than *Duox2*) and even lower levels for *Duox1* and *Nox4* (4013× and 22,595× lower than *Duox2*), whereas *Nox3* was not expressed at all by IECs. (iii) Using Western blot, we detected the DUOX2 protein in lysates of intestinal epithelial organoids derived from WT but not from *Duox2*^∆IEC^ mice (Figure 1G). Unchallenged *Duox2*^∆IEC^ mice were analyzed for their physiological phenotype. *Duox2*^∆IEC^ mice appeared healthy and showed no growth impairments or other aberrant macromorphological features. Body weight, relative weight of cecum, liver, and spleen, or length of the small and large intestine did not differ between *Duox2*^∆IEC^ and WT littermate control mice (Figure S2B). Similarly, we did not detect any alterations in the small intestinal or colonic lamina propria immune cells profile (Figure S2C) or any histological changes in the intestinal epithelial architecture (Figure S2D). To investigate whether the deletion of DUOX2 impacts epithelial ROS production, we measured ROS production by intestinal organoids, and ROS production was drastically reduced in DUOX2-deficient organoids (Figure 1H). High levels of ROS can be toxic to IECs, and we therefore measured the proliferative capability of intestinal organoids. DUOX2-deficient intestinal organoids grew faster than WT organoids (Figure 1I). Since we noted high DUOX2 levels, specifically in the apical epithelium and crypt (Figure 1A), we investigated whether cell death (apical) or proliferation (crypt) was altered in *Duox2*^∆IEC^ mice. Deletion of epithelial DUOX2 did not impact apoptosis, but higher numbers of proliferative (Ki67^+^) IECs were detected in the crypts of *Duox2*^∆IEC^ compared to WT mice (Figure 1J). The increased proliferation did not result in changes in epithelial architecture, i.e., villus length or crypt depth (Figure S2D). In summary, these data suggest that ablation of epithelial DUOX2 does not induce obvious systemic physiological alterations but stimulates intestinal epithelial proliferation.

### 3.2. Deletion of DUOX2 in the Intestinal Epithelium Alters the Mucosal Microbiome

**Figure 2:**
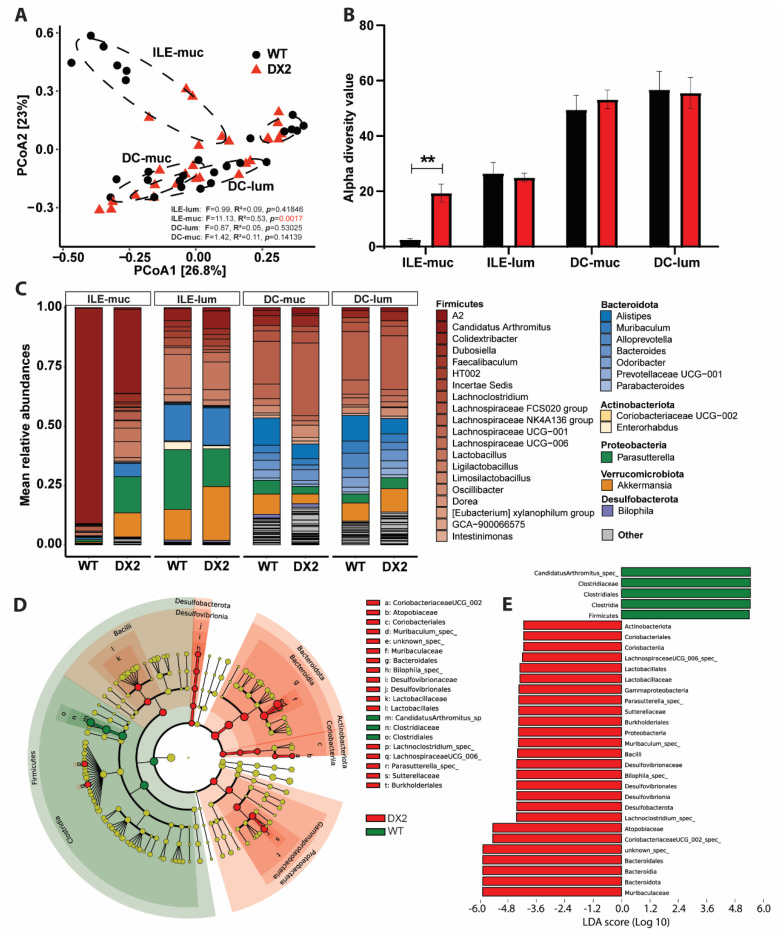
**Ablation of epithelial DUOX2 shapes the mucosal microbiome**. Paired mucosal and luminal samples from the small intestine and colon of WT and *Duox2*^∆IEC^ mice underwent 16S rRNA microbiome analysis. (**A**) Principal coordinate analysis revealed the different composition of the mucosal but not in the luminal microbiome in WT and *Duox2*^∆IEC^ mice (WT: ILE−muc *n* = 6, ILE−lum *n* = 6, DC−muc *n* = 6, DC−lum *n* = 9. DX2: ILE−muc *n* = 6, ILE−lum *n* = 6, DC−muc *n* = 7, DC−lum *n* = 8). (**B**) Alpha diversity (within sample diversity) using the Inverse Simpson metric. (**C**) Taxonomic overview on species level color−shaded by phylum. (**D**,**E**) Linear discriminant analysis (LDA) effect size (Lefse) of the ileum mucosa microbiome. (**D**) The cladogram depicts the phylogenetic distribution of differential taxa. (**E**) Differential taxa ranked by LDA. ILE = ileum, DC = distal colon. muc = mucosal, lum = lumen. ** *p* < 0.01.

ROS have antibiotic properties and are highly reactive; thus, ROS have a very short half-life and range. As DUOX2 is strongly expressed by the apical epithelium, the interaction site with the intestinal microbiome, we hypothesized that ablation of epithelial DUOX2-mediated ROS production (Figure 1A) might impact the composition of the associated microbiome. We, therefore, sampled different segments (ileum and distal colon) within the intestine and different sites (mucosa and lumen) within each segment of unchallenged *Duox2*^∆IEC^ and WT mice and surveyed their microbiome compositions. Loss of epithelial DUOX2 did not affect the composition of the luminal microbiome in the ileum or distal colon, but the mucosal microbiome differed significantly in the ileum (*p* = 0.0021) but did not differ significantly in the distal colon (*p* = 0.14509) (Figure 2A). The ileum mucosa-associated microbiome of unchallenged *Duox2*^∆IEC^ mice showed an increased alpha diversity (Figure 2B), i.e., the number and distribution of microbial taxa in a sample. Loss of epithelial DUOX2 altered the abundances of several bacterial taxa with main reductions in the relative abundance of Th17 cell-inducing Segmented filamentous bacteria (SFBs, alternatively termed *Candidatus Savagella* or *Candidatus arthromitus*) and corresponding expansions in several taxa of the Proteobacteria (Pseudomonadota) phylum (Figure 2C,D). Altogether, some of the microbiome alterations, i.e., increased diversity and reduction of Th17 cell-inducing SFBs, suggest an anti-inflammatory environment in the mucosa of *Duox2*^∆IEC^ mice. In contrast, other alterations, i.e., expansion of Proteobacteria, could also indicate a rather pro-inflammatory environment as some members of the Proteobacteria phylum have been linked to inflammation. Thus, further studies are required to elucidate the role of microbiome dysbiosis, e.g., gnotobiotic or microbiome transfer experiments.

### 3.3. Duox2^∆IEC^ Mice Are Less Susceptible to DSS Colitis and Develop an Altered Fecal Microbiome

**Figure 3:**
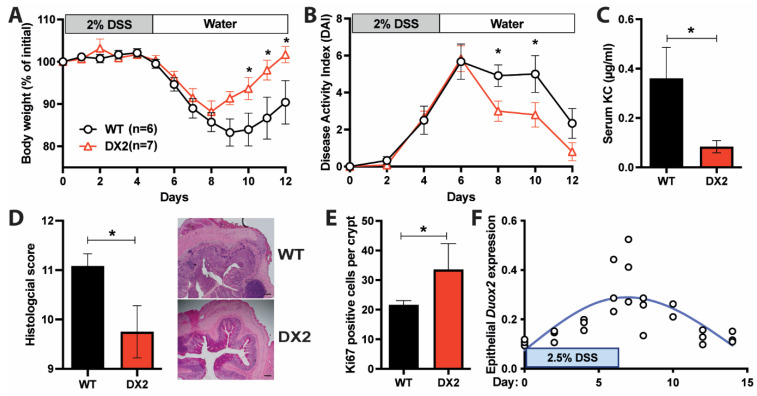
**Loss of DUOX2 in the intestinal epithelium protects from colitis**. (**A**) Body weight loss of WT and *Duox2*^∆IEC^ mice during DSS-induced colitis (WT *n* = 6, DX2 *n* = 7). (**B**) Disease activity index (DAI) consists of stool consistency, fecal blood occurrence, and body weight loss (WT *n* = 6, DX2 *n* = 7). (**C**) Serum KC/CXCL1 (pro-inflammatory cytokine) levels as determined by ELISA (WT *n* = 6, DX2 *n* = 7). (**D**) Histological score of H&E-stained colon sections, including representative images. The scale bar represents 50 µm (WT *n* = 6, DX2 *n* = 7). (**E**) Increased epithelial proliferation in *Duox2*^∆IEC^ compared to WT mice after challenge with DSS as measured by Ki-67^+^ cells per colon crypt (WT *n* = 6, DX2 *n* = 7). (**F**) Epithelial *Duox2* expression follows disease activity during colitis induction and recovery. RNAseq data [34] of WT mice treated with 2.5% DSS for 7 days and additional recovery for 7 days. *Duox2* expression was normalized to the epithelial marker gene *Cdh1*. The trendline is indicated in blue (*n* = 3). * *p* < 0.05.

As *Duox2*^∆IEC^ mice display an increased epithelial proliferation, which could lead to improved regenerative response, and harbor an altered potentially anti-inflammatory microbiome, we next investigated whether deletion of epithelial DUOX2 impacts susceptibility to acute intestinal inflammation. When *Duox2*^∆IEC^ mice and their WT littermates were challenged with 2% DSS to induce acute colitis, *Duox2*^∆IEC^ mice lost significantly less body weight compared to WT littermates and recovered more quickly, even reaching their initial starting weight by day 12 (Figure 3A). The disease activity index (DAI), a measure of intestinal inflammation comprised of body weight loss, stool consistency, and fecal blood occurrence, showed lower values for days 8–12 in *Duox2*^∆IEC^ mice, thus confirming their ameliorated disease course (Figure 3B). Additionally, *Duox2*^∆IEC^ mice displayed lower serum levels of the pro-inflammatory cytokine KC/CXCL1 as measured by ELISA (Figure 3C). Evaluation of Hematoxylin and Eosin (H&E)-stained colon sections demonstrated a reduced histological score consisting of transmural inflammation, crypt hyperplasia, epithelial injury, polymorphonuclear and mononuclear cell infiltrates in *Duox2*^∆IEC^ mice (Figure 3D). In line with the findings of unchallenged mice (Figure 1J), we detected higher numbers of proliferative (Ki67^+^) IECs in the crypts of *Duox2*^∆IEC^ mice compared to WT littermate controls (Figure 3E), indicating an improved regenerative response. Using publicly available RNA sequencing data [34], we investigated the *Duox2* expression during the disease course in the mucosa of a separate WT mouse cohort, which received 2.5% DSS for 5 days and then recovered. In these WT mice, epithelial *Duox2* expression increased along with the inflammation and then gradually decreased again during recovery and mucosal healing (Figure 3F). In summary, our data demonstrates that deletion of epithelial DUOX2 protects from acute DSS-induced colitis potentially via an improved regenerative response and mucosal healing.

**Figure 4:**
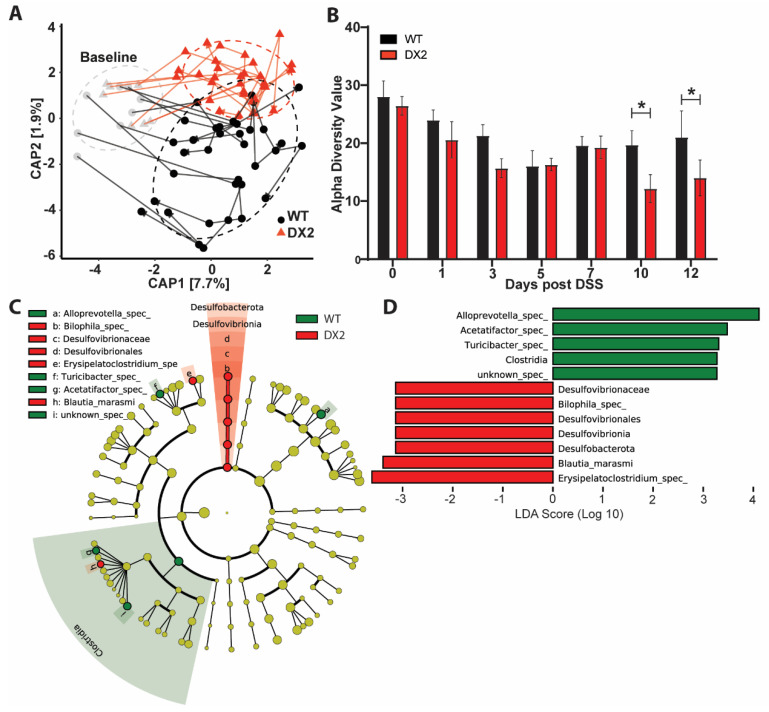
**Under inflammatory stress, *Duox2*^∆IEC^ mice develop an altered fecal microbiome**. (**A**) Constrained analysis of principal coordinates revealed differential developmental transitions in fecal microbiomes of WT and *Duox2*^∆IEC^ mice during DSS−induced colitis (WT *n* = 8, DX2 *n* = 7). (**B**) Reduced alpha diversity (Inverse Simpson metric) in *Duox2*^∆IEC^ mice at the late stage of DSS−colitis. (**C**,**D**) Lefse analysis of the merged day 7–12 microbiomes. (**C**) The cladogram depicts the phylogenetic distribution of differential taxa. (**D**) Differential taxa ranked by LDA. * *p* < 0.05.

To assess whether loss of epithelial DUOX2 impacts the fecal microbiome under acute intestinal inflammation, we analyzed the microbiome composition by 16S amplicon sequencing of fecal samples that were collected from all mice throughout the DSS colitis experiment. Confirming the data from unchallenged mice (Figure 2A), at day 0 before the administration of DSS into the drinking water, we could not detect any differences in microbiome composition between *Duox2*^∆IEC^ and WT littermate mice (Figure 4A). However, during the DSS-induced colitis and recovery, the microbiomes of *Duox2*^∆IEC^ mice and their WT littermates diverged with time and differed significantly from each other at day 10 and day 12 (*p* = 0.0325) (Figure 4A). At days 10 and 12, *Duox2*^∆IEC^ mice had slightly reduced alpha diversity (Figure 4B), and during the late inflammatory phase (days 7 to 12), few bacterial taxa were differentially abundant in *Duox2*^∆IEC^ mice compared to WT littermates (Figure 4C,D). Notably, the deletion of epithelial DUOX2 led to a reduction of the relative abundances of *Alloprevotella* and *Turicibacter*, while the relative abundance of *Bilophila* was increased. Taken together, the fecal microbiome drastically changed during acute intestinal inflammation, and our data suggests that a lack of epithelial DUOX2 may be selected for an altered fecal microbiome under inflammatory conditions.

### 3.4. DUOX2-Microbiome Interactions Are Dysregulated in Patients with Intestinal Inflammation

**Figure 5:**
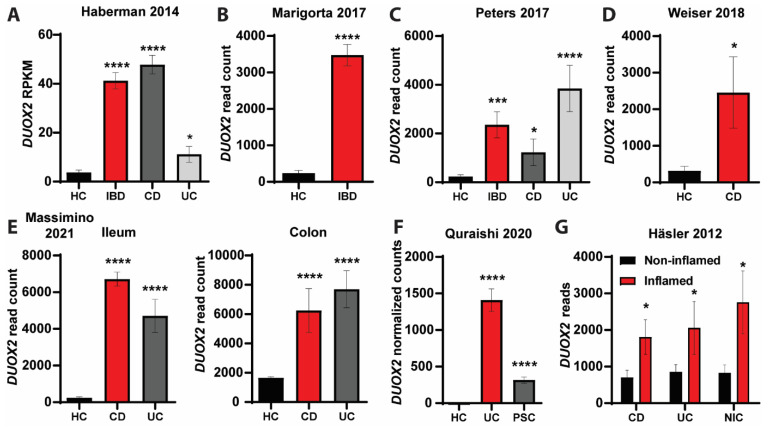
***Duox2* expression is dysregulated in the mucosa of patients with intestinal inflammation**. HC = healthy controls, CD = Crohn’s disease, UC = Ulcerative colitis, PSC = primary sclerosing cholangitis, NIC = non-IBD colitis. Note that y-axes in the panels show raw and normalized read counts; thus, data should only be compared between conditions within a single study. (**A**) *DUOX2* expression in HC (*n* = 42) and pediatric CD (*n* = 174) or UC (*n* = 38) patients and combined as IBD (*n* = 212) [41]. (**B**) *DUOX2* expression in HC (*n* = 35) and pediatric IBD patients (*n* = 210) [40]. (**C**) *DUOX2* expression in HC (*n* = 60) and CD (*n* = 42) or UC (*n* = 32) patients and combined as IBD (*n* = 74) [39]. (**D**) *DUOX2* expression in HC (*n* = 11) or CD patients (*n* = 21) [38]. (**E**) *DUOX2* expression in ileum and colon biopsies of HC (*n* = 143/86 for ileum/colon) and CD (*n* = 751/63) or UC (*n* = 133/70) patients [37]. (**F**) *DUOX2* expression in HC (*n* = 40) and UC (*n* = 40) or PSC (*n* = 40) patients [36]. (**G**) *DUOX2* expression in inflamed and non-inflamed mucosa of patients with CD, UC, or NIC [35]. *n* = 4–8 per group. * *p* < 0.05, *** *p* < 0.001 and **** *p* < 0.0001.

**Figure 6:**
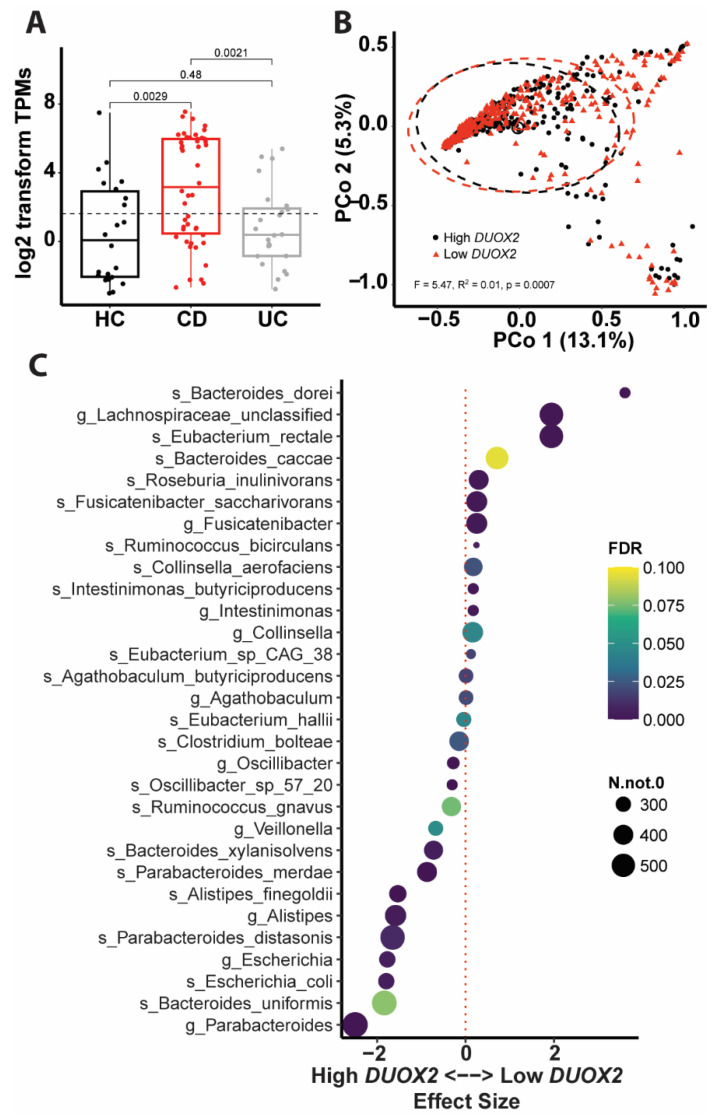
**Dysregulated DUOX2-microbiome interactions in IBD patients**. Paired mucosal *DUOX2* expression and fecal microbiome data [42] were used for an interaction analysis. (**A**) *DUOX2* expression was increased in CD (*n* = 79) and UC (*n* = 49) compared to HC (*n* = 29). Samples from CD and UC patients were divided into high and low *DUOX2* expression groups based on the median (dotted line). (**B**) Principal coordinate analysis revealed an altered fecal microbiome in high *DUOX2* expression IBD patients. (**C**) Differential taxa in high versus low *DUOX2* expression samples. Significance (FDR—false discovery rate) is color-coded, and the symbol size indicates the number of taxa reads.

By analyzing publicly available expression data of various clinical studies [35,36,37,38,39,40] derived from ileum and colon tissue samples of inflammatory bowel disease (IBD) patients, we found that *DUOX2* expression was upregulated in the mucosa of IBD patients compared to healthy controls (HC) (Figure 5 and Figure 6A). *DUOX2* expression was upregulated in both main IBD subtypes (CD, UC) and irrespective of age in young (very early onset, pediatric) and adult IBD patients. *DUOX2* expression was also upregulated in non-IBD inflammatory conditions such as primary sclerosing cholangitis (PSC) (Figure 5F) or non-IBD colitis (Figure 5G), indicating that changes in mucosal DUOX2 seem to be triggered by inflammation in general or even could be a causative trigger of the inflammation, yet further experiments are needed to disentangle these effects. Notably, *DUOX2* expression was only upregulated in the inflamed mucosa of IBD patients but not in the non-inflamed mucosa (Figure 5G).

We further aimed to investigate the interactions between DUOX2 and the microbiome under inflammatory conditions in IBD patients. To that end, we evaluated paired intestinal mucosal *DUOX2* expression and associated gut microbiome composition data of IBD patients from HMP2 [32]. DUOX2 expression was increased in the intestinal mucosa of CD and UC patients compared to HC (Figure 6A), thus corroborating findings from the other clinical cohorts (Figure 5). Next, irrespective of disease type, all samples were divided into high and low *DUOX2* expression groups based on the median *DUOX2* expression (Figure 6A). Beta diversity analysis of the fecal microbiome data revealed a significant difference (*p* = 0.0007) in the bacterial community structure between high and low *DUOX2* expression groups (Figure 6B). In the high DUOX2 expression group, the relative abundance of *Escherichia coli*, of which some strains are pathogenic, whereas others do not cause disease, along with *Alistipes finegoldii*, which has been associated with tumor formation, was increased. In comparison, the abundances of the anti-inflammatory short-chain fatty acid butyrate producers *Roseburia inulinivorans*, *Eubacterium rectale,* and *Fusicatenibacter saccharivorans* were decreased. However, it should be noted that with 16S data, strain differences cannot be resolved and need to be further investigated using, e.g., culturomics or metagenomics. Moreover, the relative abundance of several Bacteroides and Parabacteroides species was altered (Figure 6C). Thus, overall, these changes indicate a dysbiotic and pro-inflammatory microbiome in IBD patients with high *DUOX2* expression.

## 4. Discussion

We generated *Duox2*^∆IEC^ mice lacking DUOX2 specifically in IECs and analyzed their physiological responses under unchallenged and inflammatory conditions to determine the role of epithelial DUOX2 for intestinal homeostasis, host-microbiome interactions, and inflammatory susceptibility. Epithelial deletion of DUOX2 did not cause an immune phenotype under basal unchallenged conditions but increased epithelial proliferation and altered the mucosal microbiome. *Duox2*^∆IEC^ mice were protected from acute experimental colitis, which coincided with boosted epithelial regenerative responses and changes in the fecal microbiome composition in the late stages of the inflammation. Finally, we revealed that DUOX2 expression was increased in the mucosa of IBD patients, and patients with high DUOX2 expression levels also had an altered pro-inflammatory microbiome.

### 4.1. Location Matters—Epithelial DUOX2 Shapes Intestinal Homeostasis

Several studies have demonstrated the role of DUOX2 in pathogen defense and barrier function of the intestinal mucosa [9,13,15,43]. These studies either used in vitro models or *Duoxa*^−/−^ mice with a combined inactivation of DUOX1 and DUOX2 in the entire body. As DUOX2 carries out pleiotropic functions in various tissues of the body and different cell types, for example, production of the T3/T4 hormones in the thyroid, *Duoxa*^−/−^ mice are severely hypothyroid with delayed growth and development [44]. In contrast, the overall physiology of *Duox2*^∆IEC^ mice lacking DUOX2, specifically in IECs, was unaltered compared to WT littermates. *Duox2*^∆IEC^ mice displayed proper growth and a normal body size since DUOX2 remains functional in other tissues, such as the thyroid. Furthermore, *Duox2*^∆IEC^ mice with a specific deletion of DUOX2 in the intestinal mucosa were protected from acute experimental colitis, unlike *Duoxa*^−/−^ mice with systemic inactivation of DUOX1 and DUOX2, which did not display an altered susceptibility to experimental colitis [9]. Therefore, our data clearly suggests that epithelial DUOX2 may play a dichotomous role in the regulation of intestinal inflammatory responses. Very recently, *Duoxa*^∆IEC^ mice lacking both DUOX maturation factors A1 and A2 only in intestinal epithelial cells have been described, and these mice demonstrated a metabolic phenotype that was not seen in whole-body deficient mice [45], thus supporting our findings of the importance of epithelial DUOX2. Further studies are required to elucidate the precise function of DUOX2 in other cell types for intestinal homeostasis and disease susceptibility.

### 4.2. Reciprocal Interactions between Epithelial DUOX2 and the Intestinal Epithelium Microbiome

DUOX2 is regulated by the microbiome [10]. In turn, DUOX2-produced ROS have antibiotic properties, and therefore, ablation of DUOX2 could affect microbiome composition. We did not detect any changes in the luminal but in the mucosal microbiome of *Duox2*^∆IEC^ mice. ROS are highly reactive with an extremely short half-life [46]. Therefore, the effective range of ROS produced by DUOX2 in the apical epithelium may be limited and could potentially explain the restricted effect on the mucosal microbiome. Loss of epithelial DUOX2 in *Duox2*^∆IEC^ mice increased alpha diversity of the ileum mucosa microbiome mainly by reducing the abundance of SFBs, the main colonizer of the ileum mucosa. SFBs were considered strictly anaerobic but can tolerate up to 1 to 2.5% oxygen concentrations [47]. They may even be able to counteract oxidative stress using catalase [48], allowing them to colonize the mucosal compartment and even directly associate with the intestinal epithelium, where the oxygen tension is highest [49]. In the absence of epithelial DUOX2, oxygen tension of the mucosal compartment may be reduced, and other bacterial species could colonize this niche. This hypothesis is supported by the increased microbiome diversity in the ileum mucosa of *Duox2*^∆IEC^ mice. Furthermore, ablation of DUOX2 reduced ROS production and, therefore, bactericidal effects so that other more sensitive bacteria without appropriate detoxification mechanisms could expand into the mucosa. The overall increased diversity in the ileum mucosal microbiome of *Duox2*^∆IEC^ mice and taxonomic signature with decreased levels of Firmicutes and simultaneous increases of Bacteroides/Prevotella species are all features of intestinal inflammation. They can also be detected in IBD patients [1,50].

### 4.3. How Does Deletion of Epithelial DUOX2 Protect from Intestinal Inflammation?

*Duox2*^∆IEC^ mice were protected from acute experimental colitis. Several alterations in intestinal physiology may contribute to the reduced inflammatory susceptibility. DSS colitis is a microbiome-dependent experimental model as antibiotic treatment ameliorates disease development [51]. DSS-challenged *Duox2*^∆IEC^ mice had decreased levels of *Alloprevotella,* and members of the Prevotellaceae family aggravated the DSS colitis outcome [52]. Potentially, the absence of DUOX2 ROS production allows for the colonization of other bacteria, which can inhibit the growth of Alloprevotella. *Blautia*, which showed an increase in *Duox2*^∆IEC^ mice, could be a candidate bacterium due to its antibacterial activity against other microorganisms [53,54]. Levels of *Blautia* are reduced in CD patients, supporting their anti-inflammatory function [55].

The DSS colitis model also depends on mucosal injury due to the direct toxicity of DSS towards epithelial cells and appropriate regenerative responses to recover from the damage to the intestinal barrier. In line with this regenerative concept, *Duox2*^∆IEC^ mice had higher numbers of proliferative IECs in colonic crypts, and DUOX2-deficient intestinal organoids displayed increased growth, indicating improved regenerative responses and mucosal healing in the absence of epithelial DUOX2. Excessive ROS production causes DNA damage and cell death [56], which would counteract mucosal healing. Notably, it was demonstrated that in the presence of a dysbiotic microbiota, DUOX2 potentiates intestinal tumorigenesis, thus supporting the role of DUOX2 in epithelial proliferation and its dysregulation in cancer pathogenesis [43].

Although not specifically addressed in our study, DUOX2-generated H_2_O_2_ may also have additional effects on the immune response beyond its antimicrobial properties. H_2_O_2_ is an important messenger of various intracellular signaling pathways. Blocking H_2_O_2_ production could, therefore, potentially contribute to the lower levels of the pro-inflammatory cytokine KC in *Duox2*^∆IEC^ mice since ROS are required for NF-κB activation and CXCL10 production [57,58].

Overall, multiple mechanisms could underly the anti-inflammatory effect caused by the deletion of epithelial DUOX2. Further experiments are required to ultimately disentangle the cellular and molecular framework linking DUOX2 and intestinal inflammation.

### 4.4. Clinical Relevance of DUOX2

Although DUOX2 has not yet been described as an IBD risk gene by classical GWAS, two recent studies associated loss-of-function mutations of DUOX2 with preclinical hallmarks of disturbed microbiome-immune homeostasis with IBD manifestation [15,16]. In mice, epithelial deletion is protected from experimental colitis. We speculate that these differential differences may be driven by the presence of pathogens in humans that are lacking in SPF mice. Further experiments are needed to clarify this issue. By analyzing transcriptomics data from mucosal biopsies of human IBD patients from multiple clinical studies, we found increased *DUOX2* expression irrespective of age or type of intestinal inflammation (Figure 5 and Figure 6A), thus corroborating *DUOX2* as a risk gene for IBD and related inflammatory diseases. Furthermore, we identified microbiome alterations in IBD patients with high DUOX2 expression that may contribute to disease development (Figure 6B,C). In IBD patients with high *DUOX2* expression, levels of *Escherichia coli* and *Alistipes finegoldii* were increased. *Escherichia coli* is a prototypical pathobiont found in virtually everyone in the absence of disease. However, under certain conditions, for example, after antibiotic therapy, an abundance of *Escherichia coli* can increase and contribute to disease development, including intestinal inflammation [59]. Lipopolysaccharide is a component of the cell membrane of Gram-negative bacteria such as *Escherichia coli* and induces DUOX2 expression in vitro [10]; thus, the high *DUOX2* expression in IBD patients could be directly triggered by the increased abundance of *Escherichia coli. Alistipes finegoldii* promotes tumor formation [60,61], and its high abundance in *Duox2*^∆IEC^ mice is in agreement with the increased proliferation of DUOX2-deficient intestinal epithelial cells. The high levels of *Alistipes finegoldii* and epithelial proliferation appear beneficial to resolve a mucosal injury and acute inflammation, yet under chronic conditions, it could lead to colon cancer. Interestingly, under certain pro-inflammatory conditions (in TLR4-deficient mice), lack of DUOX2 indeed promotes intestinal carcinogenesis [43]. This phenomenon depends on microbiome dysbiosis; however, whether *Alistipes finegoldii* is causally involved remains elucidated. Finally, the abundance of *Roseburia inulinivorans*, *Eubacterium rectale*, and *Fusicatenibacter saccharivorans* all were reduced in IBD patients with high *DUOX2* expression. All these bacteria [62,63,64] produce the short-chain fatty acid butyrate, an anti-inflammatory mediator with pleiotropic effector mechanisms that showed beneficial prospects in clinical intervention studies for IBD [18,65]. However, although associations between DUOX2 status and changes in the microbiome in IBD patients suggest a role in disease pathogenesis, direct functional studies are needed to clarify this issue.

Our findings indicate that interactions between the microbiota and host epithelial DUOX2 contribute to healthy mucosal homeostasis and that dysregulation of epithelial DUOX2 and ROS production alters these homeostatic interactions with consequences for inflammatory susceptibility. It is important to note that ongoing inflammation can exhaust cellular metabolism and thereby cause nutritional stress, which, among others, also leads to mitochondrial oxidative stress and ROS production [66]. Thus, it will be important to consider the nutritional status of the individual and the inflamed tissue or stressed cell in future analyses. In our current study, the mice were fed a normal chow diet ad libitum, and food consumption was routinely monitored during colitis for ethical reasons; thus, we do not expect global nutritional stress, but local effects in the inflamed tissue remain to be investigated. Further efforts, such as colitis models under gnotobiotic conditions with defined microbial backgrounds and microbiome transplantations, induced conditional DUOX2 inactivation, e.g., via switchable CRE recombinases or pre-/probiotic supplementations and interference experiments, are needed to delineate the precise molecular framework of microbial control of DUOX2 expression and the feedback regulation on the mucosal microbiome function, to enable then the development of novel therapeutic strategies for the treatment of intestinal inflammation.

## Data Availability

The 16S amplicon sequencing data are accessible through the European Nucleotide Archive (ENA, https://www.ebi.ac.uk/ena accessed on 22 June 2023) under the accession number PRJEB63207. Additional data supporting the findings of this study, as well as all codes used to generate the bioinformatic analyses, are available from the corresponding author upon reasonable request.

## Supplementary Materials

The following supporting information can be downloaded at: https://www.mdpi.com/article/10.3390/antiox12101889/s1, Figure S1: Expression of NOX family members along the mouse intestinal tract and isolated IECs; Figure S2: Phenotyping of unchallenged *Duox2*^∆IEC^ mice; Table S1: List of primers used in this study.
