# Supplementary material for: Epithelial Dual Oxidase 2 Shapes the Mucosal Microbiome and Contributes to Inflammatory Susceptibility
Source: Antioxidants (Basel). 2023 Oct 21;12(10):1889. doi: 10.3390/antiox12101889 (PMC10603924; doi:10.3390/antiox12101889)
Supplement: Supplementary file 1 [file antioxidants-12-01889-s001.zip › antioxidants-2622645-supplementary.pdf]

Supplemental Information

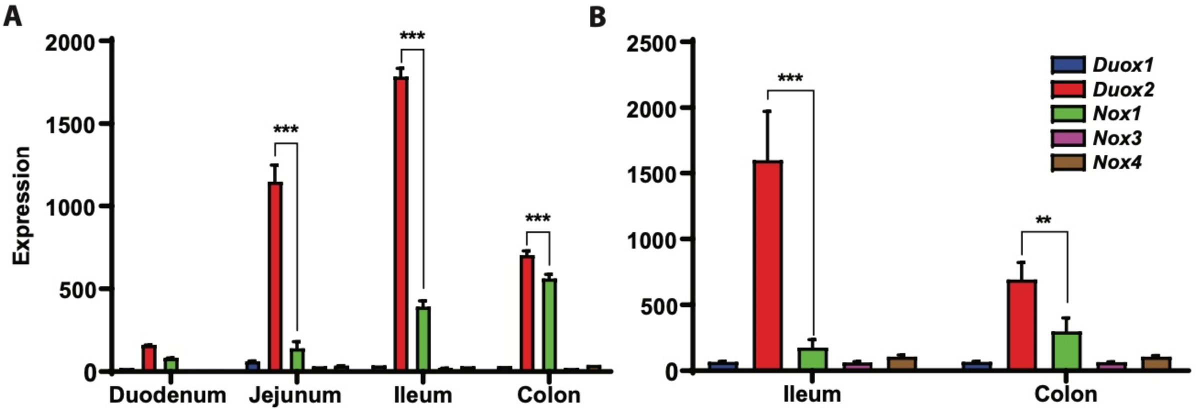

Figure S1: Expression of NOX family members along the mouse intestinal tract and isolated IECs. Expression data for non-fractionated tissue [1] and for isolated IECs [2] were retrieved from published microarray studies and analyzed for NOX family members. ** p<0.01 and *** p<0.001 FDR-corrected p values of moderated t test (for details see the original publications).

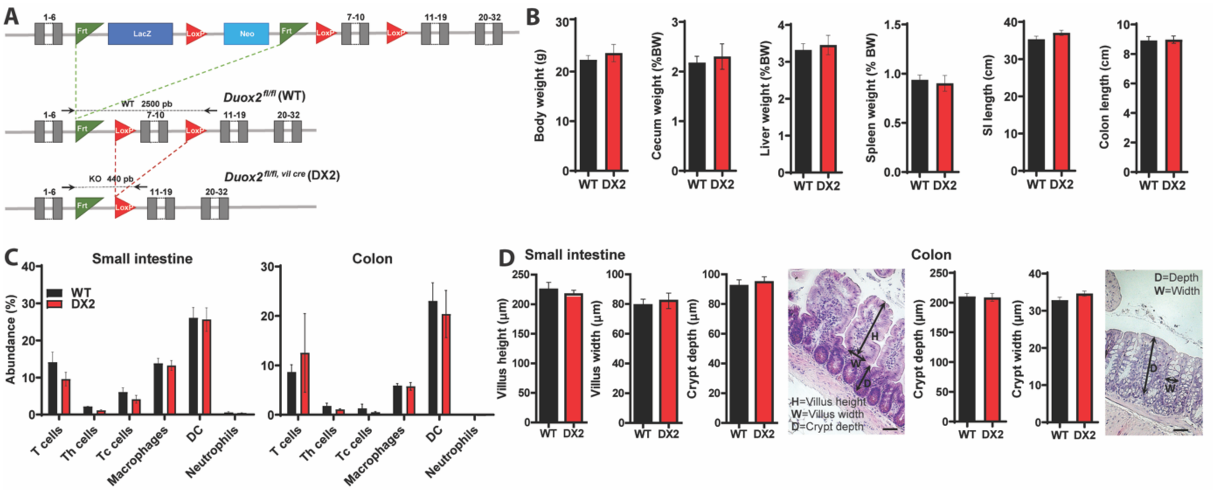

Figure S2: Phenotyping of unchallenged *Duox2$^{\Delta IEC}$* mice. A) Strategy for generating *Duox2$^{\Delta IEC}$* mice. IEC-specific DUOX2-deficient mice were generated by crossing *Duox2* floxed (*Duox2*$^{fl/fl}$) mice with Villin::CRE transgenic mice expressing CRE recombinase under the control of the IEC-specific *Villin1* promoter, resulting in specific conditional deletion of *Duox2* exons 7-10 in IECs and a non-functional DUOX2 protein due to a premature stop codon. **B)** Body and organ measures of *Duox2$^{\Delta IEC}$* and littermate control mice. Deletion of DUOX2 in IECs did not impact any of the observed organs or body weight. BW = body weight, SI = small intestine. **C)** Immune cell profile in lamina propria from small intestine and colon of *Duox2$^{\Delta IEC}$* and littermate control mice. Th = $CD4^+$ T helper cells, Tc: $CD8^+$ cytotoxic T cells. **D)** Intestinal epithelial architecture of *Duox2$^{\Delta IEC}$* and littermate control mice. Villus height and width along with crypt depth of small intestine samples, and depth and width of colonic crypts were quantified in H&E-stained intestinal sections. The scale bar in the representative images corresponds to 50 µm.

Table S1. List of primers used in this study.

**Sequence 5'-->3'**	**Gene**	**Organism**
Forward -CCCACGTTACCATTTCCATCA	*Duox1*	Mouse
CATCTGCATAGCTGGCTGGA		
ACGCAGCTCTGTGTCAAAGGT	*Duox2*	Mouse
TGATGAACGAGACTCGACAGC		
ATCCTCACAATTGTTCCCATT	*Nox1*	Mouse
TGACAACCTTGGTAATCACGA		
CCCTTTGGTACAGCCAGTGAAGAT	*Nox2*	Mouse
CAATCCCGGCTCCCACTAACATCA		
CGACGAATTCAAGCAGATTGC	*Nox3*	Mouse
AAGAGTCTTTGACATGGCTTTGG		
GGATCACAGAAGGTCCCTAGCAG	*Nox4*	Mouse
GCGGCTACATGCACACCTGAGAA		
GGCTGTATTCCCCTCCATCG	*Actb*	Mouse
CCAGTTGGTAACAATGCCATGT		

Supplementary References

1. Larsson, E.; Tremaroli, V.; Lee, Y.S.; Koren, O.; Nookaew, I.; Fricker, A.; Nielsen, J.; Ley, R.E.; Bäckhed, F. Analysis of Gut Microbial Regulation of Host Gene Expression along the Length of the Gut and Regulation of Gut Microbial Ecology through MyD88. *Gut* **2012**, *61*, 1124–1131, doi:10.1136/gutjnl-2011-301104.

2. Sommer, F.; Nookaew, I.; Sommer, N.; Fogelstrand, P.; Bäckhed, F. Site-Specific Programming of the Host Epithelial Transcriptome by the Gut Microbiota. *Genome Biol* **2015**, *16*, 62, doi:10.1186/s13059-015-0614-4.
